# Inferring Tie Strength from Online Directed Behavior

**DOI:** 10.1371/journal.pone.0052168

**Published:** 2013-01-02

**Authors:** Jason J. Jones, Jaime E. Settle, Robert M. Bond, Christopher J. Fariss, Cameron Marlow, James H. Fowler

**Affiliations:** 1 Medical Genetics Division, University of California, San Diego, La Jolla, California, United States of America; 2 Political Science Department, University of California, San Diego, La Jolla, California, United States of America; 3 Data Science, Facebook, Inc., Menlo Park, California, United States of America; University of Namur, Belgium

## Abstract

Some social connections are stronger than others. People have not only friends, but also *best* friends. Social scientists have long recognized this characteristic of social connections and researchers frequently use the term *tie strength* to refer to this concept. We used online interaction data (specifically, Facebook interactions) to successfully identify real-world strong ties. Ground truth was established by asking users themselves to name their closest friends in real life. We found the frequency of online interaction was diagnostic of strong ties, and interaction frequency was much more useful diagnostically than were attributes of the user or the user’s friends. More private communications (messages) were not necessarily more informative than public communications (comments, wall posts, and other interactions).

## Introduction

Some social connections are stronger than others. People have not only friends, but also *best* friends. They distinguish close friends from mere acquaintances. Social scientists have long recognized this characteristic of social connections and researchers frequently use the term *tie strength* to refer to this concept [1], [2]. However, online friendships (as defined by social networking sites such as Facebook and Twitter) are often considered to either exist or not exist and the continuum of tie strength is ignored [3], [4], [5]. One’s best friend and a long-forgotten, one-time classmate are grouped together under one ambiguous label of “friend.” The ability to determine which online friends represent strong ties in real, face-to-face relationships; which represent weak ties; and to know which measurements are successful proxies for such real-world tie strength could enable researchers to use online data to study face-to-face social networks [6]. This, in turn, would help social scientists and other practitioners to delve deeper into the vast quantities of data generated by the social web [7].

Several efforts have been successful in inferring tie strength from technologically-mediated communication. Tie strength can be estimated by measuring the network structure of mobile phone calls [8], the reciprocity of calls made between two mobile phone users [9], the number of tweets exchanged between Twitter users [10], network data on LinkedIn [5] and the similarity of musical tastes as measured by Last.fm [11].

In this manuscript, we determine how real world tie strength may be inferred from easily measurable online behavior and demographics. We focus our analysis exclusively on Facebook, because of its place as the most popular social networking website and its integration into numerous other highly-trafficked websites. We surveyed a group of Facebook users and asked them to name their closest friends in real life (see Materials and Methods). With ground truth established in this manner, we constructed a predictive model based on the number and nature of online interactions between survey-takers and their real world friends. The model’s success at discriminating closest friends from not-closest friends validates the use of online behavior data as a proxy measure for tie strength in real world relationships. Furthermore, our method can be used to produce a composite, quantitative estimate of tie strength for any two individuals.

## Results

All of the features included in the dataset (see Table 1 for descriptions) had at least some discriminative power to distinguish closest from non-closest friends. Table 2 contains the mean and standard error of the mean for each feature for closest and not-closest pairs and the Spearman rank correlation of each feature with the closeness target variable. Table 3 shows pairwise correlations between each of the features. Notably, there is significant positive overlap between these features. For example, people who “like” each other’s posts are also more likely to comment on each other’s posts and tag one another in photos.

**Table 1:** The names and descriptions of features used to estimate tie strength.

Feature	Description
Comments	The number of comments the survey-taker made on an object (a post, photo, link, etc.) owned by the friend.
Messages	The number of private messages (similar to email, but sent through facebook.com) the survey taker sent to the friend. Messages are visible only to the sender and recipient.
Wall Posts	The number of posts the survey-taker made on the friend’s Facebook.com wall. Wall posts are visible to the friend’s friends.
Likes	The number of times the survey-taker liked an object owned by the friend.
Photo Tags	The number of times the survey-taker tagged the friend in a photo posted on facebook.com.
Same Photo	The number of photos in which both the survey-taker and the friend were tagged (regardless of who performed the tagging).
Pokes	The number of times the survey-taker “poked” the friend through facebook.com.
Family Edges	The number of times the survey-taker requested the friend add to their profile a familial relationship to the survey-taker.
Event Invites	The number of times the survey-taker invited the friend to an event through facebook.com.
Same Gender	A binary variable that is 0 if the survey-taker and friend are of different genders or 1 if the same gender.
Same Employer	A binary variable that is 1 if the survey-taker and friend include the same employer in their facebook.com employment history and 0 otherwise.
Same School	A binary variable that is 1 if the survey-taker and friend ever attended the same school according to their facebook.com academic history and 0 otherwise.
Group Invites	The number of times the survey-taker invited the friend to join an online interest group.
Age Difference	The absolute value of the age difference between the survey-taker and friend.

**Table 2:** Descriptive statistics of the feature variables for Closest Friend dyads and Not Closest Friend dyads.

	Closest Friends		Not Closest Friends		
Feature	Mean	SEM	Mean	SEM	r
Comments	37.51	2.16	1.99	0.27	0.66
Messages	27.37	3.62	0.64	0.14	0.60
Wall Posts	7.01	0.53	0.32	0.04	0.60
Likes	22.25	1.74	1.83	0.30	0.59
Photo Tags	11.42	1.16	0.29	0.05	0.57
Same Photo	5.89	0.66	0.15	0.03	0.50
Pokes	10.05	1.80	0.14	0.05	0.31
Family Edges	0.21	0.02	0.02	0.005	0.25
Event Invites	0.56	0.05	0.19	0.02	0.21
Same Gender	0.69	0.02	0.55	0.02	0.15
Age Difference	4.96	0.39	8.08	0.51	-0.14
Same Employer	0.04	0.001	0.01	0.002	0.11
Same School	0.33	0.02	0.28	0.02	0.05
Group Invites	0.07	0.02	0.04	0.01	0.04

The final column contains the Spearman rank correlation of each feature with the Closest Friend target variable. Interaction counts represent the total interactions in the six months prior to an online survey that asked users to identify their closest friends in real life.

**Table 3:** Pairwise correlations between feature variables.

	Comments	Messages	Wall Posts	Likes	Photo Tags	Same Photo	Pokes	Family Edges	Event Invites	Same Gender	SameEmployer	Same School	Group Invites	Age Diff
Comments	1.00	0.38	0.43	0.65	0.31	0.27	0.18	0.28	0.23	0.07	0.08	0.06	0.06	-0.05
Messages		1.00	0.18	0.35	0.14	0.12	0.22	0.10	0.07	−0.05	0.00	−0.01	0.08	0.01
Wall Posts			1.00	0.45	0.34	0.30	0.16	0.13	0.15	0.02	0.05	0.06	0.03	−0.07
Likes				1.00	0.25	0.27	0.22	0.16	0.11	0.03	0.07	0.05	0.03	−0.04
Photo Tags					1.00	0.72	0.09	0.07	0.27	−0.01	0.07	0.07	0.02	−0.05
Same Photo						1.00	0.06	0.06	0.22	0.03	0.09	0.10	0.00	−0.06
Pokes							1.00	0.02	0.04	−0.05	0.00	0.01	0.00	−0.04
Family Edges								1.00	0.08	0.12	0.02	−0.01	0.00	−0.03
Event Invites									1.00	0.01	0.01	0.10	0.07	−0.03
Same Gender										1.00	0.02	0.02	0.01	0.00
Same Employer											1.00	0.01	0.05	−0.02
Same School												1.00	0.05	−0.20
Group Invites													1.00	−0.02
Age Diff														1.00

The primary technique we use to predict closeness throughout this article is logistic regression. Logistic regression has the benefits of being easily implemented and easily interpreted. The models we produce operate on dyad-level data (in other words, each row corresponds to a pair of individuals in the network who have some kind of relationship). The probability generated by these models can be interpreted as the probability that the two persons in the dyad are closest friends. We view this value as a good estimate of tie strength – higher probabilities correspond to closer ties and lower values correspond to weaker ties.

**Figure 1:**
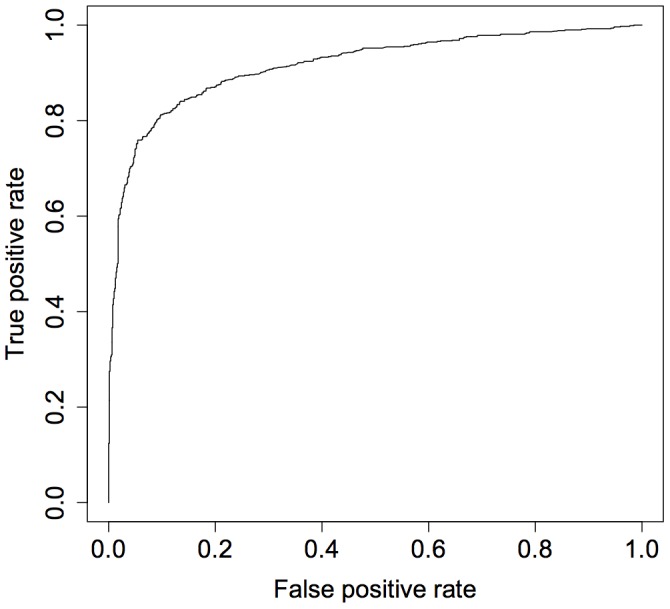
ROC curve for the additive model. The area under the ROC curve was 0.92.

We first examined an additive logistic regression model including all of the available features. The model (and all subsequent models) were evaluated through five-fold cross validation. This model achieved an accuracy of 84%. By comparison, a null model with random guessing would achieve an accuracy of 50% (95% CI 48% to 52%). The confusion matrix is presented in Table 4 and Figure 1 depicts the receiver operating characteristic (ROC) curve for this model. The area under the ROC curve was 0.92.

**Table 4:** Confusion matrix for the complete additive model.

	*Closest*	*Not Closest*
*Predicted Closest*	582	47
*Predicted Not Closest*	207	742

Columns represent ground truth and rows represent predicted values.

The value and confidence interval for each coefficient in the additive model trained on all data are listed in Table 5. The coefficients for comments, messages, wall posts, photo tags, appearing in the same photo, pokes, family members, and same-sex friendships were all positive and significant, in spite of the fact that there is substantial multicollinearity in these measures (see Table 3). Notably, traditional measures that rely on shared history (same employer, same school) or similarity in attributes (similarity in age and gender) contributed little to the prediction model.

**Table 5:** Coefficient estimates, standard errors, and 95% confidence interval for each coefficient in a model that regresses a “closest friend” indicator on the variables shown.

	*Coefficient*	*Standard Error*	*Lower 95% CI*	*Upper 95% CI*
*Intercept*	−1.873	0.154	−2.182	−1.577
***Interactions***				
*Comments*	0.039	0.011	0.019	0.062
*Messages*	0.111	0.023	0.070	0.160
*Wall Posts*	0.360	0.066	0.239	0.495
*Likes*	−0.018	0.009	−0.035	0.001
*Photo Tags*	0.183	0.040	0.111	0.269
*Same Photo*	0.303	0.098	0.132	0.507
*Pokes*	0.086	0.041	0.022	0.176
***Shared Characteristics***				
*Family Edges*	0.899	0.297	0.360	1.520
*Event Invites*	0.032	0.087	−0.132	0.209
*Same Gender*	0.818	0.157	0.514	1.129
*Age Difference*	−0.018	0.007	−0.032	−0.006
*Same Employer*	1.182	0.618	−0.001	2.466
*Same School*	−0.224	0.166	−0.553	0.099
*Group Invites*	0.032	0.139	−0.269	0.305
*N*	1578			
*Deviance*	1181.8			
*Null Deviance*	2187.6			

**Figure 2:**
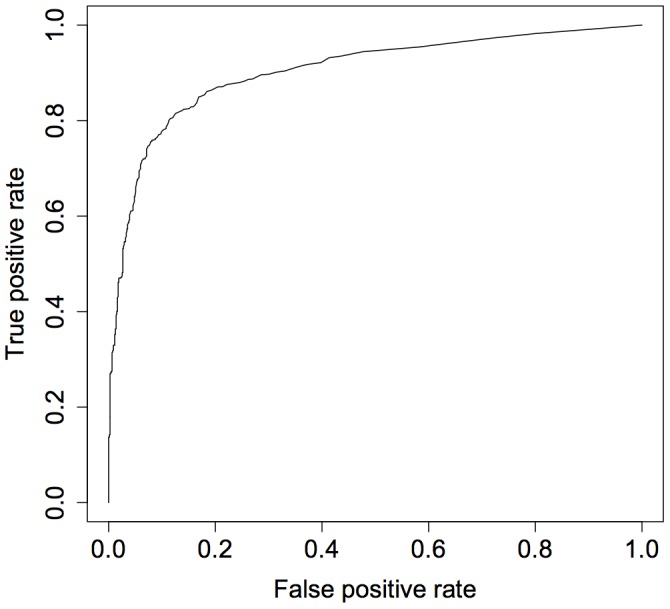
ROC curve for the Summed Interactions-only model. The area under the ROC curve was 0.90.

We can simplify the model to an extreme degree and still obtain good classification performance. We constructed a model based on one feature – the sum of all interactions observed between the users. (Specifically, Summed Interactions = Comments+Messages+Wall Posts+Likes+Photo Tags+Pokes+Event Invites+Group Invites.) This model achieved an accuracy of 82%. Figure 2 depicts the receiver operating characteristic (ROC) curve for this model. The area under the ROC curve was 0.90.

We also wished to examine whether measurement of public communication between persons is more, less, or equally diagnostic of tie strength as private communication. To this end, we compared two models based solely on one feature each. The public model is based only on wall posts. Wall posts are notes from one user to another that are visible to other users of Facebook. The private model is based on messages. Messages are notes from one user to another that are visible only to the sender and recipient.

There is very little difference in performance for models based on the two variables. For a model based solely on wall posts, accuracy is 76% and the area under the ROC curve is 0.82. For a model based solely on messages, accuracy is 75% and the area under the ROC curve is 0.81. There is no evidence that private information is a better source than public information for determining which online friendships also exist in the real world.

### Estimating Closeness with Support Vector Machines

The ability to distinguish closest from non-closest friendships is robust across types of classifier. We employed the svm() method from the R package *e1071* to train a support vector machine classifier on the full dataset. We tuned the hyper-parameters *gamma* and *cost* on random subsets of 300 dyads to find optimal values of 0.1 and 1.0 respectively. Using these values, we trained and tested a binary classifier using 5-fold cross-validation.

The classifier achieved an accuracy of 69% and a value of 0.73 for the area under the ROC curve. Performing support vector machine regression provided better results than classification. The regression was given the value 0 for non-closest friend data rows and the value 1 for closest friend rows. The output of the regression could (and did) stray outside the 0–1 band, but it is not necessary to interpret these values as probabilities. Nevertheless, using a criterion of 0.5 on the output of the support vector machine regression produced a model with accuracy 79%. The area under the ROC curve was 0.90, similar to the value of 0.92 obtained with logistic regression.

### Estimating Closeness with Random Forests

Another classification method designed to do well with high-dimensional data is the random forest approach. We employed the randomForest( ) method from the R package *randomForest.* As before, we trained and tested a binary classifier using 5-fold cross-validation.

The classifier achieved an accuracy of 86% and a value of 0.92 for the area under the ROC curve. These results are nearly identical to the results from logistic regression. Unlike logistic regression, random forest models do not place a value on a coefficient per feature. However, it is possible to extract the relative importance the model placed on each feature. The top four features important to the random forest model were Messages, Photo Tags, Comments and Wall Posts. This is further evidence that directed online behaviors are good indicators of close friendships.

## Discussion

Facebook interactions reveal strong ties. The more Alice interacts with Bob on Facebook, the more likely Alice is to name Bob as her closest friend. One can estimate tie strength simply by counting easily measurable online behaviors.

Naïve to these results, one might have argued that strong ties would be *less* likely to interact with each other on Facebook. Strong ties are more likely to see each other often in person, and strong ties likely have many other means to communicate – by phone, texting, through mutual friends, etc. Contrary to this view, we suspect our methods achieve success due to *media multiplexity*.

Media multiplexity refers to the idea that different communication media are not necessarily substitutes for each other; instead, the more two people use one medium to interact, the more likely they are to interact using other media as well [12]. Media multiplexity has been observed for email, phone, instant messaging and in-person contact [13]. In this work, we demonstrate that Facebook interactions are a good proxy measure for real world tie strength, and the advantage of Facebook interactions is that they are easily measurable compared to offline interactions.

In work similar to the current study, Kahanda and Neville [14] examined a set of Facebook users at Purdue who used the “Top Friends” application. This application allowed users to nominate a few friends as top friends, supplying a plausible set of self-labeled strong ties. Our results are consistent with Kahanda and Neville’s results. First, both studies confirm that online interactions (called transactional features in [14]) do most of the work in separating strong and weak ties. Second, both studies agree that attributes of the two users (e.g. gender, whether they attended the same school) provide some predictive power, but are dwarfed by the interaction data. The overall success of both classifiers was similar – the area under the ROC curve performance metric was 0.87 in Kahanda and Neville’s full model and 0.92 for the full model presented here.

One feature no previous work on tie strength has examined is the number of Facebook messages sent from one user to another. Messages are on the narrowest end of the visibility continuum on Facebook since they can only be seen by the sender and receiver. Wall posts are on the other end of the continuum. Wall posts are notes from one user to another, but are also visible to all the *friends* of sender and receiver.

We hypothesized that the greater intimacy afforded by private communication would mean that message counts outperform wall post counts as indicators of strong ties. However, we found no evidence this was the case. The frequency of public wall posts and the frequency of private messages correlate with the target variable of close friendship to the same magnitude, and regressions based on each separately perform equally well.

This is a result that should be useful for both users and providers of social media services. It suggests that it is not critical to have information about private communication behavior in order to characterize the likelihood that two users are closely connected in the real world. Thus, social media applications should be able to respect users’ desire for private communications and still be able to distinguish close ties from weak ties. For example, a third-party Facebook application would have no need to access private messages between users to characterize tie strength. On the Twitter platform, publicly available tweets containing at-mentions are likely to satisfactorily characterize tie-strength, and it should be unnecessary for Twitter applications to access direct messages (which are only visible to the sender and receiver).

Given that real world strong-tie connections are those most likely to transmit norms and behavior change [6], the method presented here suggests that we can identify and possibly target these relationships for a wide variety of health and other positive interventions that could yield substantial multiplier effects as they spread from person to person to person.

### Limitations

One limitation of the current study was that subjects were asked to name their closest friends and could respond with any text response. This necessitated matching records based on names entered as free response data. We failed to find a match in roughly half of cases due to survey-takers not typing full names, misspelling names or naming people with no Facebook account. If these failures to match cause data to be missing in a non-random way, then our results might not generalize to all Facebook users and their friends. We have no reason to believe our data is missing in any systematic way, however.

Another limitation was due to the choice of target variable. We chose to build a binary classifier with the goal of placing a probability on the likelihood two people were closest friends. The classifier works well for this task, and we infer that the output of the classifier also provides good information regarding tie strength in general. It may be the case that the resolution of the measure is not uniform over the entire spectrum of tie strength, however. Specifically, our method for identifying a user’s number one friend may be better at this than the related task of correctly ordering the user’s friends from closest to least close. Often, when dealing with online social graphs, we are seeking to winnow the vast tree of all existing “friendship” connections to a smaller subset of meaningful real-world connections, so a rough ordering is generally acceptable.

### Conclusion

We used online interaction data to successfully identify real-world strong ties. Ground truth was established by asking users themselves to name their closest friends in real life. We found the frequency of online interaction was diagnostic of strong ties, and interaction frequency was much more useful than attributes of the user and the user’s friends. More private communications (messages) were not necessarily more informative than public communications (wall posts). We encourage others to use interaction counts when possible to characterize the strength of relationships.

## Materials and Methods

### Participants

Written informed consent was obtained from all participants and the study was approved by and carried out under the guidelines of the Human Research Protections Program at the University of California, San Diego. 1656 users of Facebook.com completed surveys regarding their friendships. For the purposes of this study, only the responses of 789 users were appropriate for use (see below).

Participants were recruited through ads placed on Facebook.com. The ads asked Facebook users to take a short survey. The ads were targeted to English-speaking users.

Survey takers had a mean age of 29 and median age of 24 (SD = 14). 69% of survey takers were female. 96% were from the United States; 1% were from Great Britain or Canada and the remaining respondents were from other countries.

In a random sample of 500,000 Facebook users, the mean age was 28 and median 25 (SD = 13). 45% of users were female. 16% were from the United States; 5% were from Great Britain or Canada and the remaining respondents were from other countries.

While the survey-takers are not a perfect random sample of the global population nor of Facebook users, the sample is more diverse and representative than the typical convenience sample of undergraduates at a particular U.S. university.

### Procedure

We established ground truth by fielding four surveys asking Facebook users to name their closest friends. The four surveys differed in the number of close friends the survey-taker was asked to name (1, 3, 5 or 10). Each survey began with the following prompt:

*Think of the people with whom you have spent time in your life, friends with whom you have a close relationship. These friends might also be family members, neighbors, coworkers, classmates, and so on.*

*Who are your closest friends?*

We concentrated our effort on identifying closest friends. We constructed a list of positive examples by pairing each survey respondent with the first friend named in response to the first prompt. Thus, closest friends were defined as dyads including Person A (the survey-taker) and Person B (the first name generated by the survey-taker when prompted to name his closest friends).

The surveys began running in late October, 2010. As of January 14, 2011, the number of users having started a survey was 1656. The number of surveys unusable because the Facebook ID of the survey-taker was not stored was 32. The number unusable because the survey-taker did not provide the last name of the named friend was 89. The number unusable because the survey-taker did not provide the first name of the named friend was 0. The number unusable because the name of the friend provided could not be matched to the name of a Facebook friend of the survey-taker’s was 736. Thus, 789 survey-taker/closest-friend dyads were available as positive examples.

We created an equal number of negative examples. A negative example consisted of a survey-taker and one of his Facebook friends who was not named as the first closest friend. To create these dyads, a random Facebook friend was chosen for each survey-taker excluding the one friend named closest. This process results in the negative examples being drawn with uniform probability over the continuum of friendship “closeness” excluding only the most-close friendship position (that is, it could include any friend from the least close to the second-most close from amongst all the survey-takers friends). Thus, the resulting training set consists of 789 closest-friend dyads and 789 non-closest-friend dyads.

### Constructing the Dataset

Table 1 shows the features we hypothesized would be diagnostic in categorizing dyads as closest-friends vs. non-closest-friends. With the exception of the demographic variables (*Same Gender, Same School, Same Employer* and *Age Difference*), the remaining features are integer values representing the number of times the survey-taker directed the named action at his friend. The counts are over the six-month interval preceding the end of the survey period: 7/14/2010 to 1/14/2011. For example, if the value of *Messages* was 12 for one dyad, then the survey-taker sent 12 messages to the friend over the six months examined in this dataset.

The above variables are used as features to predict the value of a binary target variable: *Is Closest Friend?* The target variable is 1 if the survey-taker has named the other person in the dyad as his closest friend. The target variable is 0 if the other person in the dyad is a Facebook friend, but not the one named as the closest friend.
